# Active or passive maternal smoking increases the risk of low birth weight or preterm delivery: Benefits of cessation and tobacco control policies

**DOI:** 10.18332/tid/156854

**Published:** 2023-05-29

**Authors:** Michel-Henri Delcroix, Conchita Delcroix-Gomez, Pierre Marquet, Tristan Gauthier, Daniel Thomas, Yves Aubard

**Affiliations:** 1 Établissement Public de Santé Mentale, Association Périnatalité Recherche Information - Maternité Sans Tabac, Bailleul, France; 2 Service de Gynécologie-Obstétrique, Pôle Femme-Enfant, Centre Hospitalier d’Arras, Arras, France; 3 Service de Pharmacologie, Toxicologie et de Pharmacovigilance, Centre Hospitalier Universitaire, Limoges, France; 4 Service de Gynécologie-Obstétrique, Hôpital Mère-Enfant, Centre Hospitalier Universitaire, Limoges, France; 5 Institut de Cardiologie, Groupe Hospitalier Pitié Salpêtrière, Paris, France

**Keywords:** pregnancy, carbon monoxide, preterm birth, smoking, IGR

## Abstract

In France, maternal smoking, active or passive, remains one of the highest in Europe. At the same time, there is an increase in the number of low birth weight (<2500 g) and premature (<37 weeks of amenorrhea) newborns. The objective of this narrative review is to examine the impact of active or passive maternal smoking on birth weight or prematurity rates, and to consider the benefits of policies to stop or control smoking. This is a narrative review that analyzes and discusses the major articles published over the past 20 years regarding the role of active or passive maternal smoking on the risk of low birth weight or preterm delivery. Articles were selected using the following keywords: maternal smoking, low birth weight, preterm birth, smoking cessation, passive smoking, exhaled carbon monoxide, tobacco control policies. Active smoking is associated, in a dose-response relationship, with increased risks of low birth weight and preterm delivery. Passive smoking, mainly related to the presence of a smoking spouse, increases the risk of low birth weight and preterm birth. Our review confirmed also the benefits of smoking cessation, even in the third trimester, in reducing the risk of small for gestation age or fetal growth restriction and preterm birth. Several studies of tobacco control policies have been shown to be effective in significantly reducing maternal smoking. There is sufficient evidence to infer a causal link between active or passive maternal smoking and low birth weight or preterm delivery. This causal link is compelling and sufficient to justify intensifying efforts to promote rapid progress in tobacco control policies, with the vision of a tobacco-free generation, and smoking cessation with best practices during preconception or pregnancy.

## INTRODUCTION

Despite the organization of the Consensus Conference^1^ in 2004 with the National Agency for Accreditation and Evaluation in Health (ANAES, which then became the High Authority for Health (HAS) in 2005, i.e. French National Authority of Health), France has the highest prevalence of pregnant women who smoke in Europe^2^. According to the 2016 French national perinatal survey, 27.1% of women smoke before pregnancy, and 17.1% smoke during the third trimester^3^.

The deleterious impact of active smoking on pregnancy and/or increased risk of preterm delivery has already been well demonstrated more than twenty years ago^4-7^. Evidence from the last twenty years has been used to provide an interesting overview of the evidence published after the 2000s on the role of active or passive smoking in the increased risk of low birth weight or preterm delivery. The prevalence of neonatal morbidity associated with active and/or passive maternal smoking does not appear to have changed significantly over the last 20 years. The 2004 Surgeon General’s Report found sufficient evidence to infer a causal relationship between maternal smoking and fetal growth restriction and/or increased risk of preterm delivery^8-10^. On the other hand, the health professionals consulted by pregnant women have probably not yet sufficiently fulfilled their role of informing and managing the cessation of active or passive smoking during prenatal care.

The objective of this narrative review is to examine the impact of active or passive maternal smoking on birth weight or prematurity rates, and to consider the benefits of clinical policies to stop or control maternal smoking. This review analyzes the associations between active maternal smoking, reduced birth weight, risk of low birth weight (<2500 g) or small for gestational age; and then the associations with preterm delivery. Next, we analyzed the associations between passive maternal smoking, reduced birth weight, and the risk of preterm delivery. The benefits of cessation measures and tobacco control policies on the cessation rate of pregnant women and on neonatal indicators, are also discussed.

## DEVELOPMENTS

This is a narrative review, based on the main articles published in the last twenty years, analyzing the role of maternal smoking, active or passive, on the increased risk of low birth weight (<2500 g) or preterm delivery (<37 weeks of amenorrhea, WA). The threshold of the last 20 years was applied to provide an interesting overview of the substantial evidence published after the 2000s on the role of active or passive maternal smoking on the increased risk of low birth weight or preterm delivery.

Articles were selected using the following keywords: ‘active smoking during pregnancy’, ‘passive smoking during pregnancy’, ‘secondhand smoke during pregnancy’, ‘exhaled carbon monoxide’, ‘intrauterine growth retardation’, ‘small for gestational age’, ‘preterm birth’, ‘preterm delivery’, ‘preeclampsia’, ‘tobacco control policies’, ‘smoking cessation during pregnancy’. The databases searched were Medline, PubMed, and ScienceDirect. After reading the abstracts, only articles in English or French that could be analyzed in their full text and that had a real contributory interest in low birth weight or preterm delivery, were retained.

The selected articles provided results in the form of odds ratio (OR) or adjusted odds ratio (AOR) or difference in birth weight or gestational age in relation to active or passive maternal and/or paternal smoking status, number of cigarettes smoked per day, trimester of pregnancy, expired carbon monoxide (CO) level (ppm), maternal body mass index (BMI), birth weight (g), gestational age (WA). The risks associated with tobacco exposure during pregnancy are presented in synoptic tables in descending order.

### Active maternal smoking and reduced birth weight

Our previous research^11^, using the measurement of expired CO to objectively assess the level of fetal exposure to smoking, demonstrated that the reduction in birth weight increases as the maternal expired CO level increases. Thus, with a rate of 6 to 10 ppm, the average birth weight decrease is 350 g compared to mothers with normal exhaled CO of 5 ppm, and the average birth weight decrease is very significant (755 g) if the maternal exhaled CO is very high (20 ppm). Similarly, Bernstein et al.^12^ in 2005 discovered a linear relationship between the number of cigarettes smoked in the third trimester and the reduction in newborn birth weight. The existence of a dose time/effect relationship (reduction of approximately 27 g per cigarette smoked in the third trimester) supports the hypothesis of a causal link between active smoking exposure and birth weight reduction^12^. The findings of several studies support a causal link between maternal active smoking and low birth weight^13,14^. Table 1 presents the average decrease in birth weight related to smoking according to studies. Birth weight reduction was 332 g in women with exhaled CO levels ≥3 ppm compared to women with CO levels <3 ppm^15^ (Table 1).

**Table 1:** Maternal smoking and average birth weight reduction

*Study*	*Average birth weight reduction (g)*
**Meyer et al.**^14^ (2009)	
Cigarettes/day	
1–10	657
11–20	677
>20	735
**Gomez et al.**^11^ (2005)*	
eCO (ppm)	
6–10	451
11–20	709
>20	755
**Al-Sheyab et al.**^16^ (2016)	
Tobacco + shisha smoking	590
Shisha smoking	470
**Sabra et al.**^17^ (2018)	555
**Reynolds et al.**^15^ (2019)*	
eCO ≥3 ppm	332
**Voigt et al.**^18^ (2009)	
Average age (years)	
20	252
30	341
40	456
**Berlin et al.**^19^ (2017)	
Cigarettes/day	
1–4	228
5–9	251
≥10	262
**Ko et al.**^10^ (2014)	
Cigarettes/day	
1–10	160
11–20	175
>20	388
**Bergstra et al.**^20^ (2021)	266
**Larsen et al.**^21^ (2018)	262
**Surgeon General’s Report**^22^ (2014)*	250
**Ward et al.**^23^ (2007)	
Cigarettes/day	
≤10	86
11–20	190
>20	275
**Ribot et al.**^24^ (2014)	178
**Suzuki et al.**^25^ (2016)	125–136

* Systematic review.

### Active maternal smoking and low birth weight or fetal growth restriction or small for gestational age

Low birth weight (LBW) is defined according to the World Health Organization (WHO) as a birth weight <2500 g. Maternal smoking has been shown to increase rates of LBW or fetal growth restriction (FGR) or small for gestational age (SGA)^26^.

Maternal smoking is considered one of the leading causes and the most modifiable risk factor for LBW or FGR or SGA^17, 27-29^. Smoking during pregnancy is associated with an increased risk of LBW or SGA, with a causal link confirmed by a dose-response relationship. Many studies to date have evaluated the effects of maternal smoking on LBW, with most results showing a strong association between maternal smoking and LBW^30^, which are summarized in descending order in Table 2.

**Table 2:** Maternal active smoking and low birth weight (LBW) or fetal growth restriction (FGR) or small for gestational age (SGA)

*Authors*	*AOR (95% CI) (smokers compared with non-smokers)*
**Reynolds et al.**^15^ (2019) LBW	
eCO ≥3 ppm	OR=6.3 (1.4–27.1)
**Knight-Agarwal et al.**^31^ (2020) SGA	
BMI (kg/m²)	
18	2.66 (1.42–4.99)
19–24	3.14 (2.40–4.10)
25–29	1.92 (1.27–2.88)
30–34	2.03 (1.16–3.58)
35–39	2.37 (1.15–4.92)
≥ 40	4.51 (2.07–9.83)
**Baba et al.**^32^ (2012) SGA	2.76 (2.62–2.91)
**Wang et al.**^33^ (2020) LBW	2.4 (1.8–2.9)
**Lamm et al.**^34^ (2020) SGA	2.36 (2.34–2.38)
**Dietz**^35^ (2010) LBW	2.3 (2.3–2.5)
**Míguez et al.**^36^ (2017) LBW	OR=2.00 (1.77–2.26)
**Tong et al.**^37^ (2017) SGA	APR=2.0 (1.9–2.2)
**Meyer et al.**^14^ (2009) LBW	
Smokers 11–20 cigarettes/day	2.1 (1.7–2.5)
**Quelhas et al.**^38^ (2018) LBW	
Middle risk	1.95 (1.76–2.16)
Smokers 1–10 cigarettes/day	1.69 (1.59–1.79)
Smokers >10 cigarettes/day	2.53 (2.31–2.78
**Voigt et al.**^39^ (2006) SGA	
Cigarettes/day	
1–5	1.72 (1.71–1.73)
>21	3.50 (3.15–3.51)
**Baba et al.**^40^ (2013) SGA	
Smoking in only early pregnancy	1.26 (1.09–1.46)
Smoking throughout pregnancy	2.55 (2.43–2.67)
**Ward et al.**^23^ (2007) LBW	OR=1.92 (1.60–2.29)
**Newman et al.**^41^ (2001) FGR	
Smokers with preeclampsia	OR=1.85 (1.55–2.20)
**Inoue et al.**^42^ (2017) LBW	
Maternal and paternal smoking	1.64 (1.18–2.27)
**Blatt et al.**^43^ (2015) FGR	
Cessation after first trimester for FGR<10th	1.19 (1.13–1.24)
Cessation after first trimester for FGR<5th	1.25 (1.17–1.33)
Cessation after second trimester for FGR<10th	1.37 (1.57–1.78)
Cessation after second trimester for FGR<5th	1.83 (1.68–1.99)
Smoking throughout pregnancy FGR<10th	2.26 (2.22–2.31)
Smoking throughout pregnancy FGR<5th	2.44 (2.37–2.51)

LBW: low birth weight. SGA: small for gestational age. FGR: fetal growth restriction. AOR: adjusted odds ratio. APR: adjusted prevalence ratio. BMI: body mass index.

Smoking decreases the risk of preeclampsia but smokers with preeclampsia have a higher risk of LBW compared to non-smokers with preeclampsia^41^; however, the limited number of studies that have used a biomarker to objectively assess the level of tobacco exposure may explain an underestimation of reality^44^.

An Australian retrospective cohort study published in 2020 showed that the link between maternal active smoking and the risk of SGA is also influenced by body mass index (BMI): the smokers with a BMI ≥40 kg/m^2^ had the highest risk. Moreover, it should be noted that the use of cannabis, associated with tobacco, during pregnancy, unfortunately too often perceived by pregnant women as a safer remedy than drug prescriptions, increases also the risk of LBW^45-47^ (Table 2).

### Maternal active smoking and preterm delivery (PTD)

The increase in risk, with a dose-effect relationship, between preterm birth and maternal smoking, has been observed for several decades (Table 3). A meta-analysis published in 2000 including 20 prospective studies had perfectly demonstrated the dose-effect relationship of maternal active smoking with the risk of PTD, a 27% increase in risk^6^. This dose-effect relationship is a strong argument for a causal role of smoking in preterm birth: for light smoking, 1–10 cigarettes/day; for moderate smoking (11–20 cigarettes/day); and for intensive smoking (>20 cigarettes/day)^6^. In a study published in 2001, involving 1413811 Swedish newborns, the dose-effect relationship between maternal smoking and prematurity was also confirmed for 1–9 cigarettes/day and for ≥10 cigarettes/day^48^.

**Table 3:** Active maternal smoking and preterm delivery (PTD)

*Authors*	*OR (95% CI)*
**Tsai et al.**^50^ (2008)	
Low-risk CYP 1 A1/GSTT1 genotypes	1.6 (1.1–2.2)
High-risk CYP 1A1/GSTT1 genotypes	5.8 (2.0–21.1)
**Burguet et al.**^53^ (2004)	AOR=1.7 (1.3–2.2)
**Wang et al.**^33^ (2020)	AOR=1.6 (1.2–2.0)
**Källen et al.**^48^ (2001)	
Cigarettes/day	
<10	1.39 (1.37–1.41)
≥10	1.65 (1.62–1.68)
**Soneji et al.**^54^ (2019)	
Cigarettes/day	
Smoking stopped in first trimester	
1–9	1.16 (1.14–1.17)
10–19	1.24 (1.22–1.26)
≥20	1.30 (1.28–1.33)
Smoking stopped in second trimester	
1–9	1.42 (1.39–1.44)
10–19	1.50 (1.46–1.53)
≥20	1.58 (1.53–1.63)
**Lawder et al.**^55^ (2019)	AOR=1.41 (1.37–1.44)
**Moore et al.**^51^ (2016)	
Smoking until second trimester	AOR=1.21 (1.19–1.24)
Smoking throughout pregnancy	AOR=1.70 (1.60–1.80)
**Baba et al.**^32^ (2012)	AOR=1.30 (1.25–1.36)
**Shah et al.**^6^ (2000)	1.27 (1.21–1.33)
Cigarettes/day	
1–10	1.25 (1.12–1.38)
11–20	1.38 (1.23–1.55)
>20	1.31 (1.19–1.45)
**Diguisto et al.**^56^ (2020)	AOR=1.21 (1.19–1.24)

AOR: adjusted odd ratio.

The arguments for inferring a causal link with smoking are the pre-existing relationships, stability, dose-effect and duration effect. These arguments are found in the studies summarized in Table 3 with adjustment of the odds ratio on other risk factors for preterm birth.

Different studies have found that certain genotypes (CYP1A1 and GSTT1) interact with smoking to modify the risk of preterm birth (PTD)^49,50^. The risk of preterm birth in case of maternal smoking, is increased by 60% compared to non-smoking mothers with low-risk genotypes (CYP1A1 [Aa/aa] and GSTT1 genotypes). Maternal smoking significantly increased the risk of PTD in women with high risk CYP1A1 and GSTT1 genotypes, and particularly high in some subgroups^50^.

A retrospective cohort study, published in 2016^51^ involving 913757 single live newborns between 20 and 42 weeks, assessed the risk of preterm birth according to the duration of maternal smoking : smokers in the second trimester or throughout pregnancy, compared to non-smokers, had respectively a 21% and 70% increased risk (OR adjusted for ethnicity, education level, social protection, marital status and parity).

The risk of prematurity is highest when childbirth is associated with histological chorioamniotis, but the association with smoking is due to the fact that smoking women have bacterial vaginosis and/or premature rupture of membranes much more often than non-smokers^52^ (Table 3).

### Passive maternal smoking and its implications on birthweight and pre-term delivery

In our study published in 2005^11^, we had noted with the objective measure of paternal expired CO at delivery, that the impact of passive smoking on the reduction of birth weight was important and proportional to the level of exposure to paternal smoking. These results confirm the dose-effect relationship previously mentioned for maternal active smoking and that the impact of exposure to secondhand smoke should be better explored. Furthermore, a study published in 2020, showed that indoor parental passive smoking, linked to the presence at home of a smoking spouse, doubles the risk of low birth weight and even triples this risk when combined with high outdoor air pollution^57^ (Table 4). These results show the necessity of obtaining smoking information from both parents to evaluate the real adverse effect of passive smoking during pregnancy^58^.

**Table 4:** Passive maternal smoking and birth weight reduction (BWR) or LBW (<2500 g)

*Authors*	*BWR (g) or AOR (95% CI) for LBW*
**Lu et al.**^57^ (2020)	
Alone passive smoking	2.17 (1.09–4.33)
Both exposure to outdoor pollution	3.45 (1.27–9.39)
**Norsa’adah and Salinah**^59^ (2014)	153
	2.60 (1.60–4.16)
**Gomez et al.**^11^ (2005)	
Paternal eCO (ppm)	
6–10	62
11–20	237
>20	356
**Ribot et al.**^24^ (2014)	129
**Miguez et al.**^36^ (2020)	105
**Ward et al.**^23^ (2007)	36 (rang: 5–67)
**Leonardi-Bee et al.**^60^ (2008)	33 (range: 16–51)
	OR=1.32 (1.07–1.63)

BWR: birth weight reduction. LBW: low birth weight. AOR: adjusted odds ratio.

The dose-dependent relationship between passive smoking and preterm birth has been well confirmed, indicating the harmfulness of passive smoking on the risk of preterm delivery. This risk is particularly high when the importance of passive smoking is evaluated objectively, for example by measuring nicotine in the maternal hair (risk multiplied by 6 if the concentration of nicotine in the hair is ≥4 μg/g). The risk of premature delivery is also very high (risk multiplied by 4) when the spouse is a heavy smoker (more than 20 cigarettes per day)^61^ (Table 5).

**Table 5:** Passive smoking and preterm birth (PTB)

*Authors*	*AOR (95% CI)*
**Jaakkola et al.**^62^ (2001)	
Hair nicotine concentration ≥4.0 µg/g	6.12 (1.31–28.70)
**Rajia et al.**^61^ (2020)	
Spouse smoking >20 cigarettes/day	4.03 (1.2–13.5)
**Qiu et al.**^63^ (2014)	
Very preterm birth <32 gestational weeks	OR=1.98 (1.41–2.76)
**Cui et al.**^64^ (2016)	1.20 (1.07–1.34)
**Leonardi-Bee et al.**^60^ (2008)*	OR=1.18 (1.03–1.35)

* Meta-analysis. AOR: adjusted odds ratio.

### Effectiveness of smoking cessation

A meta-analysis published in 2009 showed that different individual interventions to reduce smoking during pregnancy (72 trials between 1975 and 2008 involving 25000 women) reduced by 6% (RR=0.94; 95% CI: 0.93–0.96) the risk of low birth weight, and by 14% (RR=0.86; 95% CI: 0.74–0.98) that of preterm birth^65^.

Studies published after this meta-analysis confirmed the benefits of quitting smoking even in the third trimester in reducing the risk of SGA or of FGR and PTB^66^. Baba et al.^22^ demonstrated a greater efficacy with intense interventions of smoking cessation, with a 19% risk reduction (RR= 0.81; 95% CI: 0.69–0.96) of low birth weight and a 16% risk reduction (RR=0.84; 95% CI: 0.71–0.99) of preterm birth. If mothers quit smoking before the end of the first trimester of pregnancy, the birth weight is the same as that of children of non-smoking mothers. If mothers continue to smoke during pregnancy, birth weight is reduced depending on the level and duration of smoking^51^.

In addition to its own deleterious impact, spouse smoking greatly increases the risk for the mother to continue smoking, up to nearly 9 times higher (OR=8.70; 95% CI: 7.39–10.20)^67^. Also, smoking cessation programs should therefore include smoking cessation management for smoking fathers at the same time as for mothers^68,69^.

The commitment of perinatal professionals to help women who smoke, pregnant or want to have a pregnancy to quit smoking, is a key contributor to the efficiency of prenatal care^70^. Quitting smoking before the third trimester removes or reduces the risk of neonatal morbidity, LBW or SGA or FGR and PTB^32,43,70^. The effectiveness of stopping exposure to maternal smoking justifies that all effective interventions be mobilized to promote smoking cessation as early as possible and throughout pregnancy^71,72^. This mobilization of resources by health professionals to stop the exposure of unborn children to tobacco smoke appears all the more necessary as the negative effects of the continuation of this exposure have been and remain underestimated (a significant percentage of women continuing to smoke without revealing it which induces biases in the results)^35,73^. Among the factors for continuation of maternal smoking is the co-consumption of tobacco and cannabis (adjusted prevalence ratio, APR=1.6; 95% CI: 1.2–2.3)^74^. This co-consumption, willingly underestimated if it is not systematically sought, has an even more deleterious impact than smoking only. A meta-analysis, published in 2013 that included 86 studies, found that behavioral interventions for pregnant women are effective in increasing rates of smoking cessation (RR=1.45; 95% CI: 1.27–1.64) as well in reducing rates of low birth weight (RR=0.82; 95% CI: 0.71–0.94) or of preterm birth (RR=0.82; 95% CI: 0.70–0.96)^75^.

### Effect of tobacco control policies on perinatal health

Several studies of tobacco control policies, taxes, and smoke-free air laws, have proven to be effective, with a significant effect on reducing maternal smoking^76,77^.

In the United States, data from pooled cross-sections of women with live births during 2000–2005 in 29 states plus New York City (n=225445) showed that a $1.00 increase in taxes and smoking bans in public places, had increased the number of smoking cessations in the third trimester by between 4 and 5% after adjusting for the other covariates^76^. In Scotland, the implementation of national smoke-free air laws had an effect on reducing the prevalence of prenatal smoking and preterm delivery^78^, further strengthening the evidence of the impact of smoke-free legislation on educational differences in birth outcomes^79^. Indeed, in Ireland, a study showed a 25% decrease in the rate of prematurity after the legislation (2003–2005) prohibiting smoking in public places^80^, while in Belgium, the rate of prematurity decreased after the smoking ban in restaurants in 2007 and after the smoking ban in bars in 2010^81^.

### Arguments to encourage tobacco control and for a tobacco-free future

Overall, active smoking during pregnancy doubles the risk of low birth weight and increases the risk of preterm birth by 21%. Passive smoking, mainly related to the presence of a smoking spouse, increases the risk of low birth weight and that of preterm birth. It should be noted that the deleterious effects of active and/or passive smoking during pregnancy described above are underestimated, due to biases closely related to under-declaring of smoking before or during pregnancy: up to 22.9% of pregnant smokers and 9.2% of non-pregnant smokers of reproductive age did not accurately disclose their smoking status^35^.

This effects synthesis of the fetal exposure to active or passive smoking during pregnancy is sufficient to infer a causal relationship between exposure to active or passive tobacco smoke and LBW or PTB. Hence, some authors have long insisted on the need to pay as much attention to the prevention of maternal exposure to passive smoking as to the prevention of active smoking^58,59^.

### Implications

According to the World Health Organization, preterm birth is a leading cause of neonatal morbidity and mortality, affecting approximately 15 million children worldwide each year, with more than one million requiring medical attention^82^. During pregnancy, maternal smoking and SHS exposure are the leading preventable causes of perinatal morbidity (LBW and/or PTB)^83^.

The burden of disease from tobacco smoke exposure is very heavy, and among the heaviest burdens are those affecting infants^84^. Smoking parents, being the main source of the child’s exposure to tobacco smoke^85^, by quitting smoking will save years of quality of life and economize in very high healthcare expenses^86^. According to the Global Burden of Disease Pediatric Collaboration study, the preterm birth complications are the second cause of death or disability adjusted life years (DALYs) in children and adolescents aged 0–19 years. In 2013, the leading causes of death among younger children (aged <5 years) were globally, after lower respiratory tract infections, preterm birth complications (742381 deaths; 95% CI: 591348–910767)^87^.

Ultimately, neonatal morbidity, limited here to low birth weight (LBW) and premature birth (PTB), linked to *in utero* exposure to smoking, active and/or passive, represents for the child both a major health inequality and a flagrant injustice^88,89^. This meta-analysis with 41 studies from North America, Europe and China had estimated that implementation of smoke-free policies was associated with reduction in the rate of preterm birth (OR= -3.8; 95% CI: -6.4% – -1.2%)^87^.

Smoke-free legislation also appears to have its effect by reducing secondhand smoke. Increasing tobacco taxation and the minimum legal age to purchase cigarettes are the most effective tools to reduce smoking prevalence and the rate of low-birth-weight babies^90^.

Reducing smoking prevalence, and SHS exposure are major goals of the WHO FCTC ratified by 181 countries. So, it is of significant concern that MPOWER policies are only fully implemented by a minority of these countries^91^.

The WHO has formulated six key tobacco control policies that participating countries need to implement, represented by the MPOWER acronym (**M**onitor tobacco use and prevention policies; **P**rotect people from tobacco smoke; **O**ffer help to quit tobacco use; **W**arn about the dangers of tobacco; **E**nforce bans on tobacco advertising, promotion and sponsorship; and **R**aise taxes on tobacco). The imperative need to avoid the exposure of the unborn child to maternal and/or paternal smoking, although already implicit in the Convention on the Rights of the Child, must be considered from the perspective of ‘human rights’^92^ and Article 9 of the WHO Framework Convention on Tobacco Control (FCTC)^93^.

The health professionals^94^ should also always provide evidence based services to really help pregnant women avoid active and passive tobacco and/or cannabis smoke. Individual actions to help people quit smoking before or during pregnancy are public health actions, which are essential to rapidly improve children’s health^95^. Clinicians have a responsibility to facilitate a safe and guilt-free environment for all pregnant women, to provide them with support, information and motivational tools adapted to enable them to stop smoking and avoid exposure to secondhand smoke. To achieve this, the means of effective action are: access to nicotine replacement therapy (NRT), other tobacco addiction treatments as well as cognitive behavioral therapies, application of smoke-free policies, media campaigns, and increased tobacco taxes^68,71,96^.

In the world, as in France, there are too many premature births and newborns with low birth weight, whose main preventable cause is maternal and/or paternal smoking^97^. All perinatal professionals should be involved in preventing, detecting active and/or passive smoking during pregnancy and, if necessary, implement appropriate clinical modalities to enable each smoking woman and/or spouse to successfully quit smoking^98^.

On the other hand, given that one of the factors playing an important role in the continuation of maternal smoking is a smoking spouse, with a risk likely 9 times higher, it is imperative to ensure paternal smoking cessation at the same time as maternal smoking cessation. Smoking cessation programs should include smoking fathers at the same time as the most vulnerable smoking mothers. Finally, to better preserve the health of unborn children, the strategy would benefit from mobilizing NGOs committed to a tobacco-free generation and a tobacco-free society by organizing the denormalization of tobacco^98,99^. Indeed, to fight effectively against the tobacco industry, which has always targeted children and adolescents, the means are known and must be mobilized^100^.

The generalization of tobacco-free pregnancy will come from joint commitment of health professionals. Researchers should be conscious of their responsibility and opportunities to engage with policymakers and media to achieve such a tobacco-free pregnancy in the future^101^. This result will arrive at best in the vision of an endgame of the tobacco epidemic^98^.

### Strengths and limitations

This narrative review has several strengths: it analyzed the consequences of active or passive smoking during pregnancy on two complications, low birth weight and preterm delivery, which directly impact the health of newborns and children. Our review also showed the benefit of management programs and tobacco control measures in reducing *in utero* tobacco exposure of children and in improving neonatal morbidity indicators. Limitations are that most publications have assessed the level of exposure to active or passive smoking during pregnancy on the basis of patients’ self-report. This is a narrative review that did not follow a systematic approach to study identification and data extraction, while we did not perform a meta-analysis.

## CONCLUSION

The analysis of the main publications of the last 20 years clearly highlights a strong relationship between smoking, active or passive, and birth weight reduction and risk of low birth weight or premature birth. Smoking during pregnancy is one of the leading preventable causes of neonatal morbidity.

These undeniable negative effects on neonatal morbidity, limited here to low birth weight and prematurity, highlight the major public health challenge of preventing active and passive smoking during pregnancy. Finally, this comprehensive strategy would benefit not only the health of children, but also all of humanity. While active and/or passive smoking during pregnancy continues to be one of the leading preventable causes of childhood morbidity worldwide, all efforts must be made to reduce active and/or passive smoking among women of childbearing age.

The consequences for the child, low birth weight or prematurity, related to his exposure *in utero* to maternal smoking, active or passive, should be analyzed in the light of human rights and the rights of the child. All means should therefore be mobilized to protect the fetus from exposure to tobacco smoke.

## Data Availability

Data sharing is not applicable to this article as no new data were created.
